# Fecal microbiota transplantation for the treatment of neurogenic rosacea: protocol of a randomized, blinded, placebo-controlled explorer study

**DOI:** 10.1080/07853890.2026.2735091

**Published:** 2026-10-14

**Authors:** Xia Zhu, Linyan Cheng, Shuxiao Li, Dongjie Guo, WanJun Guo, Xinran Du, Ran Yan, Xinhe Wang, Shaojie Yuan, Moan Chen, Weiming Zhang, Siyu Li, Fulun Li, Lei Zhang

**Affiliations:** ^a^ Department of Vascular Surgery, Yueyang Hospital of Integrated Traditional Chinese and Western Medicine, Shanghai University of Traditional Chinese Medicine, Shanghai, China; ^b^ Department of Dermatology, Yueyang Hospital of Integrated Traditional Chinese and Western Medicine, Shanghai University of Traditional Chinese Medicine, Shanghai, China; ^c^ Department of Dermatology, Shuguang Hospital Affiliated to Shanghai University of Traditional Chinese Medicine, Shanghai, China

**Keywords:** Neurogenic rosacea, fecal microbiota transplantation, gut microbiota, neurogenic inflammation, gut-skin axis

## Abstract

**Introduction:**

Neurogenic rosacea is a distinct phenotype of rosacea characterized by persistent facial erythema, flushing, burning, and stinging, and it often responds poorly to conventional therapies. Increasing evidence suggests that gut microbiota dysbiosis may be involved in the pathogenesis of NR. Fecal microbiota transplantation may provide a novel therapeutic strategy by restoring microbial homeostasis.

**Objective:**

This study aims to evaluate the efficacy and safety of FMT in patients with NR and to explore its potential mechanisms through modulation of the gut microbiota.

**Patients and Methods:**

This is a single-center, randomized, double-blind, placebo-controlled exploratory trial. Sixty patients with NR will be randomly assigned (1:1) to receive either FMT capsules or placebo capsules for 3 consecutive weeks and followed for 12 weeks. The primary outcome is the proportion of participants achieving CEA success at week 12, defined as a CEA score of 0 or 1 or a reduction of ≥2 grades from baseline. Secondary outcomes include flushing severity, symptom scores, quality of life, psychological status, objective erythema index, and safety. Exploratory analyses will assess gut microbiota composition, fecal and serum metabolomics, and neurovascular-related cytokines.

**Discussion:**

This study is the first clinical trial to explore the effects of fecal microbiota transplantation in the treatment of neurogenic rosacea. The trial may provide clinical evidence for the application of fecal microbiota transplantation as a novel therapeutic strategy for refractory neurogenic rosacea.

**Trial registration:**

Chinese Clinical Trial Registry (ChiCTR2500109957) on September 28, 2025.

## Introduction

Rosacea is a chronic, relapsing inflammatory skin disorder that predominantly affects the central face, with a global prevalence of approximately 5.5% [1]. Its clinical manifestations include persistent erythema, episodic flushing, papules and pustules, and telangiectasia. The condition significantly impairs patients’ quality of life, increases psychological burden, and limits social functioning. In recent years, neurogenic rosacea (NR) has been proposed as a clinically recognizable phenotype of rosacea [2], characterized by prominent neurosensory symptoms such as burning, stinging, and dysesthesia [3]. These patients also frequently present with neuropsychiatric comorbidities, including anxiety, depression, and migraine, suggesting a distinct neurogenic inflammatory basis [4]. Although NR may clinically overlap with erythematotelangiectatic rosacea (ETR), it is distinguished by prominent neurosensory symptoms triggered or exacerbated by factors such as emotional stress, temperature fluctuations, sun exposure, and spicy foods, disproportionate sensory discomfort relative to visible cutaneous findings, and poor response to conventional rosacea therapies. These clinical features have subsequently been incorporated as core diagnostic features in several studies of NR [3,5–7].

Current evidence indicates that the pathogenesis of NR involves dysregulation of neural, immune, and vascular functions. Abnormal activation of sensory nerves promotes the release of vasoactive neuropeptides, leading to vasodilation and neurogenic inflammation, while also enhancing vascular reactivity and cutaneous sensory sensitization. Among these mediators, the key neuropeptide calcitonin gene-related peptide (CGRP) is elevated in the serum of patients with rosacea, particularly those with NR, in whom circulating CGRP levels are positively correlated with disease severity and flushing [5,8]. Meanwhile, elevated circulating levels of pro-inflammatory cytokines, including IL-1β, IL-17, IL-8, and TNF-α, have been observed in patients with rosacea, with some of these mediators also associated with anxiety and depressive symptoms [9]. Moreover, interactions among nerve endings, vascular endothelial cells, and immune cells form a complex regulatory network that collectively drives disease progression [4,10]. However, most existing studies focus on local cutaneous mechanisms, and the upstream regulatory factors remain insufficiently understood.

In recent years, increasing attention has been paid to the role of the gut microbiota in chronic inflammatory skin diseases [11,12]. The gut microbiota contributes to disease pathogenesis by modulating systemic inflammation, immune homeostasis, and neuroendocrine signaling [13]. Patients with rosacea exhibit significant gut microbial dysbiosis [14] and are frequently associated with gastrointestinal comorbidities, including Helicobacter pylori infection, small intestinal bacterial overgrowth, and inflammatory bowel disease [15]. Disruption of gut microbiota may contribute to neurovascular dysfunction in rosacea by impairing intestinal barrier integrity, promoting the release of pro-inflammatory cytokines, and modulating neurotransmitter synthesis [16]. Additionally, alterations in microbially derived metabolites and neuroactive substances may influence sensory nerve excitability and vascular tone, thereby promoting erythema and flushing [17]. However, gut-related mechanisms are unlikely to be uniformly relevant across all rosacea phenotypes. The recently proposed skin-gut-brain axis framework suggests that gastrointestinal dysfunction and gut microbial dysbiosis may play a more prominent role in patients with marked flushing, burning, and stress-responsive symptoms than in those with predominantly local inflammatory lesions [17]. These features constitute key clinical diagnostic clues for NR. Preliminary direct evidence specific to neurogenic rosacea has also emerged. A cross-sectional case-control study involving 20 patients with NR and 14 healthy controls identified alterations in gut microbial composition and circulating metabolomic profiles, as well as significant correlations between specific bacterial taxa and circulating metabolites [7].

Fecal microbiota transplantation (FMT) involves the transfer of functional microbial communities from healthy donors into the recipient’s gastrointestinal tract to restore microbial homeostasis [18]. As one of the most effective approaches for reconstructing gut microbial diversity, FMT has been applied in various gastrointestinal and extraintestinal diseases, including inflammatory bowel disease [19], irritable bowel syndrome [20], metabolic disorders [21], diabetic peripheral neuropathy [22], and dermatological conditions [12,23]. However, despite accumulating evidence linking rosacea to gut microbiota dysbiosis, clinical studies evaluating FMT in this condition remain lacking. Therefore, we propose to conduct a randomized, double-blind, placebo-controlled clinical trial to assess the efficacy and safety of FMT in patients with NR and to explore its potential mechanisms through modulation of the gut microbiota.

## Patients and methods

### Study design and registration

This study is a single-center, randomized, double-blind, placebo-controlled exploratory clinical trial. It has been approved by the Ethics Committee of Yueyang Hospital of Integrated Traditional Chinese and Western Medicine, Shanghai University of Traditional Chinese Medicine (Approval No.: 2025-212), and has been registered with the Chinese Clinical Trial Registry (http://www.chictr.org.cn) (Registration No.: ChiCTR2500109957). A total of 60 participants are planned to be enrolled between October 2025 and October 2026. The study will be conducted in accordance with the Declaration of Helsinki, and written informed consent will be obtained from all participants prior to enrollment. The study design and protocol will be reported in accordance with the Standard Protocol Items: Recommendations for Interventional Trials (SPIRIT) statement (Supplementary Material 1). The intervention consists of a 3-week course of FMT capsules or placebo capsules, followed by a 12-week follow-up period (Figure 1). Recruitment will be conducted through outpatient posters and online advertisements on Chinese social media platforms (WeChat). Eligible participants will be screened at the dermatology outpatient clinic of the study center.

**Figure 1.**
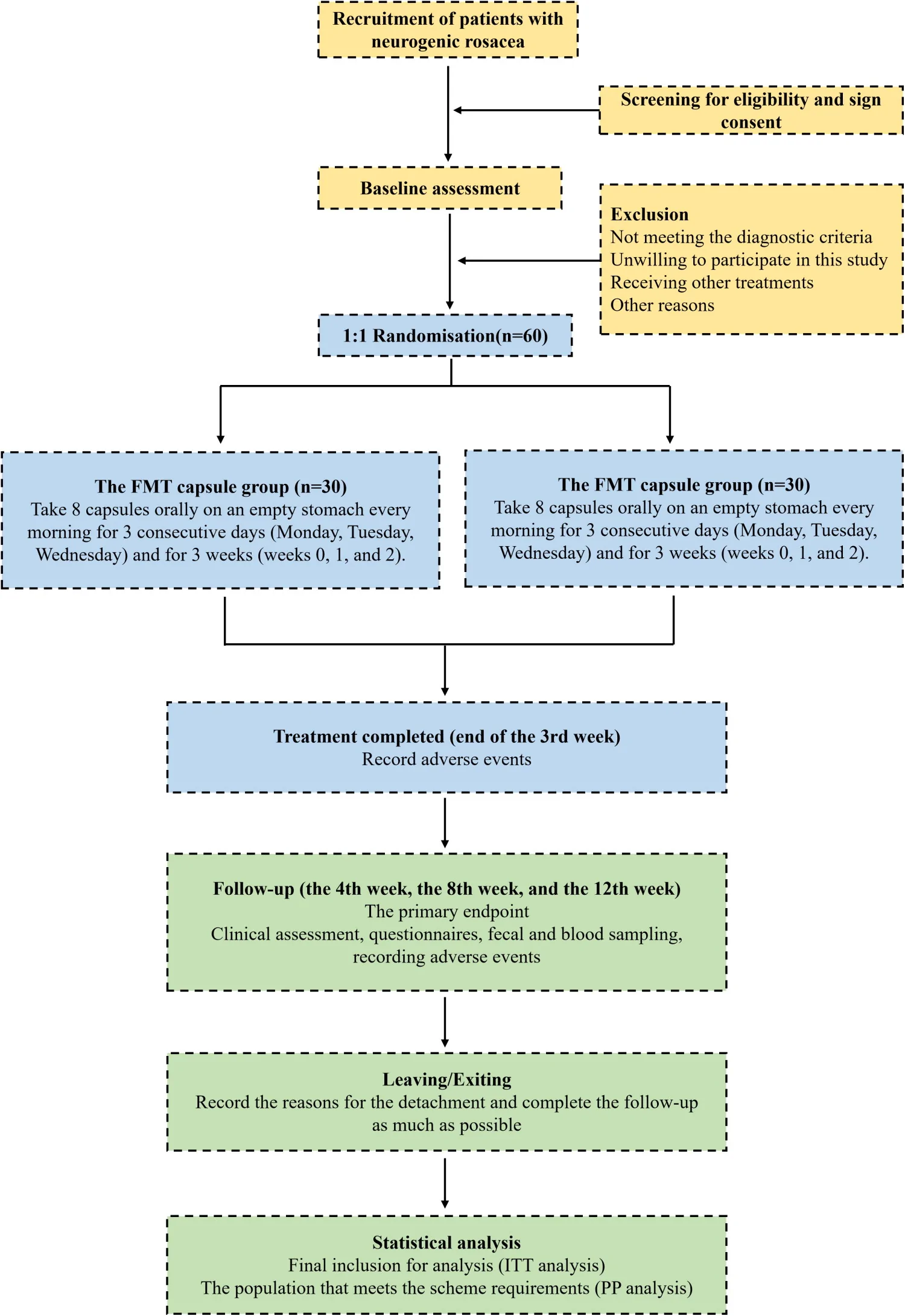
Flow chart of the trial. FMT, Fecal Microbiota Transplantation.

### Patients

A total of 60 participants will be enrolled in this study through consecutive recruitment of patients with NR attending the dermatology outpatient clinic of Yueyang Hospital of Integrated Traditional Chinese and Western Medicine. Patients who meet the inclusion and exclusion criteria will be invited to participate in the study. A clinical research coordinator will conduct preliminary screening and face-to-face interviews with potential participants to further assess their eligibility. For those willing to participate, investigators will provide a detailed explanation of the study objectives, procedures, as well as potential risks and benefits prior to enrollment. Participants will be included only after voluntarily signing written informed consent. All eligible participants who meet the inclusion criteria will undergo baseline assessments before being randomized into study groups and receiving the corresponding interventions.

#### Diagnostic criteria

The diagnosis of rosacea will be established according to the 2017 National Rosacea Society Expert Committee criteria. As internationally validated diagnostic criteria for NR are currently unavailable, the neurogenic phenotype will be identified using clinical features consistently described in previous NR studies [3,5–7]. To improve diagnostic reproducibility, eligibility for NR will be independently assessed by three experienced dermatologists, with disagreements resolved by consensus.

Diagnostic features (presence of ≥1 is sufficient for diagnosis):

1. Persistent centrofacial erythema that may periodically intensify;
2. Phymatous changes (characterized by tissue hypertrophy, presenting as skin thickening and irregular contours, most commonly affecting the nose).

Major features (presence of ≥2 suggests rosacea):

1. Flushing;
2. Papules and/or pustules;
3. Telangiectasia;
4. Ocular manifestations (including lid margin telangiectasia, blepharitis, keratitis, conjunctivitis, and keratoscleritis).

#### Eligibility criteria

1. Age between 18 and 50 years, regardless of sex;
2. Persistent facial erythema;
3. Presence of neurogenic symptoms such as burning or stinging sensations;
4. Symptoms sensitive to specific triggers, such as emotional stress, temperature fluctuations, sun exposure, and spicy food;
5. Poor response of facial erythema to conventional treatments, including antibiotics (metronidazole, doxycycline, minocycline), oral isotretinoin, and oral β-blockers;
6. Willingness to participate in the study and provision of written informed consent.

#### Exclusion criteria

1. Pregnant or lactating women, or those planning pregnancy;
2. Patients with moderate to severe phymatous or ocular rosacea;
3. Patients with severe or clinically unstable systemic diseases, including symptomatic cardiovascular disease, severe cerebrovascular or pulmonary disease, cirrhosis or other severe hepatic dysfunction, clinically significant renal impairment, serious hematological disorders, or active malignancy;
4. Patients with contraindications to FMT, including those with severe immunodeficiency (e.g., organ transplant recipients receiving immunosuppressive therapy, advanced HIV/AIDS, or congenital immunodeficiency), active or uncontrolled gastrointestinal diseases (e.g., acute intestinal obstruction, intestinal perforation, severe flare of inflammatory bowel disease, intra-abdominal abscess), severe allergic predisposition or known allergy to transplant materials, and those with known or potential high risk of infection (e.g., active chronic hepatitis B or C infection);
5. Patients with a history of severe psychiatric disorders;
6. Patients who received systemic treatment with conventional medications within 4 weeks prior to the trial or who used isotretinoin within 6 months prior to the trial;
7. Patients with other facial dermatoses (e.g., seborrheic dermatitis, dermatomyositis, systemic lupus erythematosus);
8. Patients who used antibiotics or probiotics within 1 month prior to the trial.

### Randomization and blinding

The randomization sequence will be generated by an independent statistician using GraphPad Prism 10 software, who will not be involved in participant recruitment, intervention delivery, outcome assessment, or data analysis. Participants will be randomly assigned in a 1:1 ratio to either the FMT group or the control group. The allocation results will be placed in sequentially numbered, sealed, opaque envelopes and kept by a designated individual independent of the clinical research team. This study is designed as a double-blind trial. Participants, investigators, outcome assessors, and statisticians will all remain blinded to group assignments throughout the study. FMT capsules and placebo capsules will be identical in appearance, packaging, and administration regimen to ensure proper blinding. Both types of capsules will be provided by the manufacturer and distributed weekly by a designated study nurse.

### Study interventions

All participants will undergo a 4-week washout period prior to randomization, during which any systemic treatments that may affect the gut microbiota or the course of rosacea are prohibited, including antibiotics, isotretinoin, probiotics, and other related interventions. All participants will receive standardized background care, including disease management and patient education, avoidance of known triggers (such as ultraviolet exposure, emotional stress, and spicy foods), enhanced photoprotection, and the use of low-irritation topical moisturizers. On this basis, participants will be randomly assigned to receive either FMT capsules (Patent No. ZL201920077799.2) or placebo capsules, both manufactured by Shanghai Biotecan Pharmaceuticals Co., Ltd. (Shanghai, China).

#### Donor screening and selection

Healthy stool donors aged 18–30 years with a body mass index (BMI) of 18.5–23.9 kg/m^2^ are recruited from the general population. Donors undergo a structured health questionnaire assessing medical history, medication use, lifestyle and dietary habits, travel history, and behavioral risk factors, followed by laboratory screening. Blood screening includes routine hematological testing, liver and renal function, electrolytes, C-reactive protein, and testing for transmissible infections, including viral hepatitis, human immunodeficiency virus, syphilis, Epstein-Barr virus, cytomegalovirus, SARS-CoV-2, and selected parasitic infections. Stool screening includes routine stool examination and fecal occult blood testing, together with testing for Clostridioides difficile, Campylobacter spp., Salmonella spp., Shigella spp., Shiga toxin-producing Escherichia coli, parasites, norovirus, rotavirus, SARS-CoV-2, Candida albicans, and multidrug-resistance determinants, including extended-spectrum β-lactamase and carbapenemase genes. Donors undergo repeat screening every 8 weeks to maintain eligibility.

#### FMT capsule preparation and quality control

Donated stool is collected in sterile containers and processed under aseptic conditions within 6 h of defecation. Eligible stool samples (≥100 g; Bristol Stool Form Scale types 3–5) are processed immediately or temporarily stored at 2-8 °C before processing. At least 50 g of stool is homogenized with sterile normal saline at a ratio of 1:3 and filtered. The resulting microbial suspension is centrifuged at 4 °C, after which the supernatant is removed and a lyoprotectant containing skimmed milk powder and glycerol is added. The suspension is mixed, pre-frozen, and lyophilized for 24 h, and the resulting lyophilized microbial powder is encapsulated in acid-resistant hydroxypropyl methylcellulose capsules.

Each capsule contains 0.8 g of lyophilized material. Quality control is performed for each production batch, including pathogen screening, bacterial viability testing, viable cell counting, and assessment of microbial composition for batch consistency. Capsule release requires bacterial viability of ≥70% and a viable bacterial count of ≥1 × 10^9^ CFU/g. Each production batch is tested for potential pathogens, including C. difficile, Campylobacter spp., Salmonella spp., Shigella spp., Shiga toxin-producing E. coli, parasites, norovirus, rotavirus, SARS-CoV-2, and multidrug-resistance determinants. Five percent of each batch is retained frozen for 1 year for traceability. The prepared capsules are stored at −80 °C for up to 6 months or at −20 °C for up to 3 months and transported under cold-chain conditions using dry ice or at −15 °C to −25 °C. The capsules are manufactured in a facility equipped with a biosafety level 2 laboratory and a Good Manufacturing Practice (GMP) production workshop under standardized procedures for preparation, storage, quality control, and transportation.

The placebo capsules contain skimmed milk powder, fructooligosaccharides, mannitol, and caramel food coloring, with no viable microorganisms or donor-derived microbial components. The placebo capsules will be identical in appearance and administration regimen to the FMT capsules but will not contain donor-derived microbial components. The dosing regimen consists of oral administration of 8 capsules once daily in the morning on an empty stomach for three consecutive days (Monday, Tuesday, and Wednesday), totaling 24 capsules per week, for 3 consecutive weeks (Week 0, Week 1, and Week 2), followed by a 12-week follow-up period. The capsules will be transported under cold-chain conditions and incubated at 37 °C for 10 min prior to administration. Participants are required to fast for 4 h before dosing and continue fasting for 1 h after administration. Throughout the study, participants will be instructed to maintain stable patterns of daily activity, physical activity levels, and lifestyle habits. To minimize the potential influence of dietary factors on the gut microbiota, participants will be asked to maintain their habitual dietary patterns and avoid substantial dietary changes throughout the trial [24]. Dietary intake will be assessed using 3-day food records before the visits at baseline and Weeks 4, 8, and 12, corresponding to the scheduled fecal sample collection time points. Participants will be asked to record all foods, beverages, and dietary supplements consumed during each recording period. Any substantial changes in dietary habits during the study will also be documented [25]. Participants will be prohibited from using any additional treatments for rosacea during the study. Intervention will be discontinued or adjusted based on investigator judgment in cases of serious adverse events, withdrawal of informed consent, or major protocol deviations that may affect study evaluation.

### Primary and secondary outcomes

The primary outcome is to evaluate whether modulation of the gut microbiota by FMT can improve the clinical severity of NR. This will be assessed by the proportion of participants achieving Clinical Erythema Assessment (CEA) success at Week 12^26^. CEA success is defined as a CEA score of 0 or 1 at Week 12, or a reduction of ≥2 grades from baseline.

Secondary outcomes include clinical efficacy, symptom improvement, safety, and mechanistic endpoints, as detailed below:

1. The proportion of participants achieving flushing success, defined as a reduction of ≥2 points in flushing severity score (0, clear; 1, almost clear; 2, mild; 3, moderate; and 4, severe) [26] from baseline at Week 12;
2. Change in CEA scores from baseline to Week 4 and Week 8;
3. Change in Flushing Assessment Tool (FAST) [27] scores from baseline to Week 12;
4. Change in Patient Self-Assessment (PSA) [28] scores from baseline to Week 12;
5. Change in lesion counts and Investigator’s Global Assessment (IGA) [26] scores from baseline to Week 12;
6. Change in burning, stinging, and pruritus Visual Analog Scale (VAS) [29] scores from baseline to Week 12;
7. Change in quality of life (RosaQoL, DLQI) and psychological status (DASS-42) [26] scores from baseline to Week 12;
8. Change in objective erythema index from baseline to Week 12;
9. Incidence of adverse events and serious adverse events during the study period.

In addition to the assessment of clinical efficacy and safety, exploratory analyses of the gut microbiome, fecal and serum metabolomic profiles, and circulating neuroimmune-related mediators will be performed using biological samples collected during the study. At each scheduled sampling time point, approximately 5 g of fecal samples and 10 mL of blood samples will be collected [24]:

1. To evaluate changes in gut microbiota composition and metabolic profiles before and after FMT, and to analyze the relationship between microbial engraftment and clinical efficacy;
2. To assess changes in fecal and serum metabolites before and after FMT, and to identify microbial metabolic signatures associated with disease improvement;
3. To evaluate changes in circulating neuroimmune-related mediators, including CGRP, IL-1β, IL-17, IL-8, and TNF-α.

The participant timeline is presented (Table 1).

**Table 1.** Trial schedule with data capture of patient-reported outcomes.

		Screening	Treatment			Follow-up		
Subjects	Research period	Week 0	Week 0 (±3 days)	Week 1 (±3 days)	Week 2 (±3 days)	Week 4 (±3 days)	Week 8 (±3 days)	Week 12 (±3 days)
Enrollment and baseline information	Inclusion/exclusion criteria	×						
	Informed agreement	×						
	Random allocation	×						
	Demographic features	×						
	Disease history and duration	×						
	Previous medications used	×						
	Concomitant medications review	×						
Intervention	FMT capsule		×	×	×			
	Placebo capsule		×	×	×			
Efficacy evaluation	Clinical Erythema Assessment (CEA)	×				×	×	×
	Flushing Assessment Severity Tool (FAST)	×				×	×	×
	Patient’s Self-Assessment (PSA)	×				×	×	×
	Investigator’s global assessment (IGA)	×				×	×	×
	Burning/stinging/itching (VAS)	×				×	×	×
	Quality of life (RosaQoL, DLQl)	×				×	×	×
	Psychologicalstatus (DASS-42)	×				×	×	×
Diet records	All foods, beverages, and dietary supplements		×	×	×	×	×	×
Safety evaluation	Adverse events and serious adverse events	×	×	×	×	×	×	×
Exploratory assessments	Stool sample collection	×				×		×
	Blood sample collection	×				×		×

FMT, Fecal microbiota transplantation; CEA, Clinical Erythema Assessment; FAST, Flushing Assessment Severity Tool; PSA, Patient’s Self-Assessment; IGA, Investigator’s global assessment; VAS, Visual analogue scale; DLQl, Dermatology Life Quality Index; RosaQOL, Rosacea-specific quality of life; DASS-42, Depression Anxiety Stress Scales-42.

### Data collection, management, and analysis

During the screening visit, eligibility will be assessed through face-to-face interviews and verification using the electronic medical record system to determine whether participants meet the inclusion and exclusion criteria. Prior to randomization, baseline demographic and clinical data will be collected, including age, sex, disease duration, potential triggering factors, prior treatment history, concomitant medications, and baseline severity scores of NR. All assessments will be performed using standardized rating scales to ensure consistency across different time points and participants. All outcome measures will be evaluated according to a predefined schedule (baseline, Week 4, Week 8, and Week 12). Safety data, including adverse events and serious adverse events, will be continuously recorded and monitored throughout the study period. Participants will complete patient-reported outcome measures at specified time points under the guidance of study personnel. All study data will be collected by trained assessors who remain blinded to group allocation. Data will be recorded in case report forms (CRFs) and subsequently entered into a secure, encrypted electronic database. Data entry will be performed by trained study staff and verified through double data entry to ensure accuracy.

An independent Data and Safety Monitoring Board (DSMB) will oversee study conduct and participant safety throughout the trial. The DSMB will conduct an initial safety review after the first cohort of participants has received the study intervention and will subsequently review accumulated safety data at regular intervals throughout the 12-week study period until completion of safety follow-up. Safety reviews will include the frequency, severity, and relatedness of adverse events (AEs) and serious adverse events (SAEs), suspected FMT-related infections or adverse reactions, and other clinically significant safety signals. Additional ad hoc reviews will be convened if an SAE, unexpected adverse event, or other clinically significant safety concern occurs. An SAE considered potentially related to FMT, a suspected serious FMT-related infection or adverse reaction, or any emerging safety signal indicating an unacceptable risk to participants will trigger prompt safety evaluation and temporary suspension of further study intervention pending review. Based on its review, the DSMB may recommend continuation of the trial without modification, protocol modification, temporary suspension, or early termination for safety reasons.

The primary efficacy analysis will follow the intention-to-treat (ITT) principle, with all randomized participants analyzed according to their originally assigned treatment group, regardless of treatment adherence or study discontinuation. Participants who withdraw or are lost to follow-up will therefore remain in the ITT population, with all available outcome data included in the analysis. A per-protocol (PP) analysis will be performed as a sensitivity analysis and will include participants who complete the study without major protocol deviations. Missing outcome data will be handled using multiple imputation under the assumption that data are missing at random. The imputation model will incorporate treatment allocation, baseline outcome values, available post-baseline measurements, and relevant baseline characteristics. The primary analysis based on the imputed datasets will be compared with a complete-case analysis as a sensitivity analysis to assess the robustness of the findings.

The primary outcome is the proportion of participants achieving CEA success at Week 12. CEA success is defined as a CEA score of 0 or 1, or a reduction of ≥2 grades from baseline. Differences in response rates between the two groups will be compared using the chi-square test or Fisher’s exact test, as appropriate. The treatment effect will be estimated by calculating odds ratios (ORs) with 95% confidence intervals (CIs). For secondary outcomes, continuous variables will be presented as mean ± standard deviation or median (interquartile range), depending on data distribution. Between-group comparisons will be performed using the independent samples t-test or Mann-Whitney U test. Categorical variables will be analyzed using the chi-square test or Fisher’s exact test. For repeated measures data, including CEA scores, flushing severity scores, symptom scores, and objective skin parameters, repeated-measures analysis of variance (ANOVA) or linear mixed-effects models will be applied to assess time effects, group effects, and group-by-time interactions.

Exploratory gut microbiome analyses will be performed using shotgun metagenomic sequencing of fecal samples collected at baseline and Weeks 4, 8, and 12. After quality control and removal of host-derived reads, taxonomic profiling will be performed at the species level using MetaPhlAn4, and microbial functional profiles will be characterized using HUMAnN3. Alpha diversity will be assessed using observed species richness, Shannon, and Simpson indices. Beta diversity will be evaluated using Bray-Curtis dissimilarity and visualized by principal coordinates analysis (PCoA). Differences in overall microbial community structure between treatment groups and across time points will be assessed using permutational multivariate analysis of variance (PERMANOVA), with the longitudinal repeated-measures structure taken into account and multivariate dispersion assessed as appropriate. Differential abundance analysis will be performed using ANCOM-BC2 to identify taxa showing differential changes between treatment groups over time, accounting for repeated measurements and relevant covariates, with false discovery rate (FDR) correction for multiple testing. The Wilcoxon rank-sum test and paired Wilcoxon signed-rank test will be used to assess between-group and within-group differences in microbial and metabolite features, respectively. Multiple testing across high-dimensional microbial and metabolite features will be controlled using the Benjamini-Hochberg FDR procedure. Associations between longitudinal changes in microbial taxa or functional pathways and changes in clinical outcomes, including CEA, flushing severity, and symptom scores, will be explored using Spearman’s rank correlation and multivariable association models, as appropriate. In addition, changes in fecal and serum metabolomic profiles, as well as neurovascular-related cytokines, will be analyzed to explore potential mechanisms underlying the therapeutic response to FMT.

### Sample size calculation

At present, no direct clinical trial data are available on FMT for NR, and reliable disease-specific estimates of the treatment effect are therefore unavailable for sample size estimation. Similar challenges have been encountered in previous FMT studies investigating novel extraintestinal indications. In the absence of prior efficacy estimates, these early studies have generally been conducted as exploratory or proof-of-concept trials [22] with relatively modest sample sizes, typically involving approximately 30–60 participants [12,30,31], to generate preliminary evidence and inform subsequent larger-scale studies. Given the exploratory nature of the present study, the anticipated response rates were informed by available clinical evidence on FMT in related inflammatory skin diseases. In a randomized, double-blind, placebo-controlled exploratory trial of FMT for moderate-to-severe atopic dermatitis, 84.6% and 92.3% of participants receiving FMT achieved EASI50 at Weeks 4 and 8, respectively, compared with 25.0% and 37.5% in the placebo group [12]. Because these findings cannot be directly extrapolated to NR, a more conservative response rate of 63% was assumed for the FMT group. Based on previous rosacea studies and available control-group data from related studies, a response rate of 25% was assumed for the placebo group [26,32]. Using a two-sided test with a significance level of α = 0.05 and a statistical power of 1-β = 0.80, sample size estimation was performed based on comparison of two independent proportions. The results indicate that 27 participants are required per group. After accounting for an anticipated 10% dropout rate, a final sample size of 30 participants per group was determined, yielding a total of 60 participants.

It should be noted that, because the anticipated treatment effect in this study is not based on prior direct clinical data from patients with NR, the true treatment effect may be smaller than expected. In such a scenario, the present study may be underpowered to detect a more modest between-group difference. Therefore, the study findings will also be interpreted in conjunction with the estimated treatment effect and its 95% confidence interval. The preliminary effect estimates obtained from this study will further provide a basis for sample size estimation and the design of future confirmatory clinical trials.

### Ethics and dissemination

Before the initiation of the trial, ethical approval was obtained on September 19, 2025, from the Ethics Committee of Yueyang Hospital of Integrated Traditional Chinese and Western Medicine, Shanghai University of Traditional Chinese Medicine (Approval No.: 2025-212). The study was registered on September 28, 2025, in the Chinese Clinical Trial Registry (ChiCTR2500109957; https://www.chictr.org.cn/showprojEN.html?proj=289252). This study will be conducted in strict accordance with the Declaration of Helsinki. Written informed consent will be obtained from all participants prior to enrollment. The participation of each patient will be kept confidential, and their identity will be restricted to the study center only, subject to participant consent.

Given the increasing international attention to novel therapeutic strategies for NR, the results of this study will be submitted for publication in peer-reviewed international journals and will also be presented at both national and international academic conferences.

## Discussion

NR is a distinct subtype of rosacea that remains associated with significant unmet therapeutic needs [33]. Unlike classical phenotypes dominated by papulopustular lesions or isolated vascular dilation, NR is characterized by persistent facial erythema, recurrent flushing, and prominent neurogenic symptoms, including burning, stinging sensations, and increased cutaneous sensitivity, all of which severely impair patients’ quality of life [4]. However, conventional therapies targeting classical rosacea, such as anti-inflammatory agents and vasomodulatory treatments, show limited efficacy in NR. This reduced therapeutic response is largely attributed to the fact that NR is not primarily driven by localized cutaneous inflammation or vascular abnormalities alone, but rather by dysfunction of the neuro–immune–vascular axis. This includes heightened neural sensitization, vascular hyperreactivity, and aberrant interactions with the central nervous system [33,34]. To date, no standardized treatment strategy specifically targeting the underlying pathophysiology of NR has been established [35].

Numerous clinical observations indicate that patients with NR frequently present with a range of extra-cutaneous manifestations, including anxiety, depression, migraine, irritable bowel syndrome, and other gastrointestinal dysfunctions [36–39]. These comorbidities strongly suggest that NR is not merely a localized facial condition, but rather a systemic disorder involving neuroimmune, vascular, and metabolic dysregulation [40–42]. As a key regulatory hub linking the intestinal barrier, immune system, and neuroendocrine network, the gut microbiota has the potential to exert upstream regulatory effects [22,43–45]. Previous studies have demonstrated significant differences in gut microbiota composition between patients with rosacea and healthy individuals, indicating that gut microbial dysbiosis may contribute to disease pathogenesis [14]. Further investigations focusing on the NR subtype have shown that these patients exhibit not only more pronounced alterations in gut microbial structure but also distinct changes in serum metabolomic profiles, particularly involving pathways related to neuroendocrine dysregulation [7]. Notably, microbial dysbiosis in NR may reflect a global imbalance of the gut ecosystem rather than changes in specific bacterial taxa alone [7]. Gut microbiota imbalance may influence neurovascular function through multiple mechanisms, including immune modulation, neurotransmitter regulation, and microbial metabolite signaling [46]. Fecal microbiota transplantation can comprehensively restore the structure and function of the gut microbiota and has demonstrated promising therapeutic potential in inflammatory bowel disease, metabolic syndrome, atopic dermatitis, and diabetic peripheral neuropathy [12,22,47,48]. Therefore, this study adopts a randomized, double-blind, placebo-controlled design to systematically evaluate the efficacy and safety of FMT in patients with NR. In addition, integrated microbiome and metabolomics analyses will be conducted to explore the potential mechanisms underlying its therapeutic effects.

This study is the first clinical trial to investigate FMT in the treatment of NR. Participants are diagnosed according to the 2017 criteria of the National Rosacea Society Expert Committee and are further restricted to the neurogenic phenotype, requiring the presence of persistent facial erythema, neurogenic symptoms, and inadequate response to conventional therapies. CEA is a standardized and validated clinician-reported measure that has been widely used to assess treatment response in clinical trials of rosacea. Recent randomized controlled trials evaluating neurotargeted or neuromodulatory interventions, such as gabapentin and repetitive transcranial magnetic stimulation, in patients with rosacea characterized by prominent neurovascular manifestations have also used CEA as the primary efficacy endpoint [26,28,49–51]. Therefore, the primary outcome of the present study is the proportion of participants achieving CEA success at Week 12, a CEA score of 0 or 1, or a reduction of ≥2 grades from baseline, representing a clinically meaningful improvement. Neurosensory symptoms, including burning, stinging, and pruritus, are important clinical manifestations of NR; however, there is currently no internationally accepted and validated NR-specific composite measure that comprehensively assesses these neurosensory symptoms. Accordingly, neurogenic symptoms are included as prespecified secondary efficacy outcomes in this trial to complement the assessment of improvement in the characteristic neurogenic symptoms of NR. Secondary outcomes encompass multidimensional clinical endpoints, including flushing severity scores, patient self-assessment scales, VAS scores for neurogenic symptoms, quality of life measures, and psychological assessment scales. In addition, an objective erythema index is incorporated to enhance the comprehensiveness and objectivity of efficacy evaluation. To further investigate underlying mechanisms, fecal and blood samples will be collected before and after treatment. Integrated analyses combining metagenomics, metabolomics, and neurovascular-related cytokine profiling will be performed to explore the potential biological basis of FMT in NR. During the study period, the use of any medications that may interfere with the gut microbiota, including antibiotics and probiotics, is prohibited. The use of such agents within one month prior to enrollment is defined as an exclusion criterion to minimize bias and confounding effects.

At the same time, this study has several limitations. First, as a single-center exploratory trial with a relatively small sample size, the generalizability of the findings requires further validation in larger, multicenter studies. Enrollment is also restricted to participants aged 18–50 years to maintain a relatively homogeneous study population and reduce potential confounding related to age-associated differences in gut microbiota composition, comorbidities, and concomitant medication use. However, this age restriction may further limit the generalizability of the findings to older patients with NR. Future studies with broader age ranges will be needed to determine whether the efficacy and safety of FMT are consistent across different age groups. Second, the 12-week follow-up period is relatively short and may be insufficient to fully assess the long-term durability of microbiome engraftment, the persistence of clinical benefits, and rosacea relapse. Longer-term clinical and microbiome follow-up will therefore be needed in future studies to determine the stability of microbial engraftment and the durability of the therapeutic response to FMT. In addition, there is currently no standardized FMT regimen for rosacea, including optimal dosage, frequency, and treatment duration. In this study, the treatment protocol was informed by previous applications of FMT in other skin diseases [12,52] and clinical experience, adopting a regimen of 8 capsules taken once daily for three consecutive days per week, for a total of 3 weeks. This regimen is empirical in nature and may require further optimization in future studies. Furthermore, the microbiome and metabolomics analyses in this study are primarily exploratory and aimed at identifying potential associations. Proteomic profiling is not included, which may limit a more comprehensive characterization of host molecular responses to FMT. Therefore, future mechanistic studies integrating metagenomics, metabolomics, and proteomics will be needed to further investigate the causal relationships underlying these findings and the mechanisms underlying the therapeutic effects of FMT.

Overall, this study will provide preliminary clinical evidence for the application of fecal microbiota transplantation in NR and offer new insights into the role of the gut microbiota in this disease. If the results are favorable, future multicenter validation studies will be warranted, along with further investigations into the therapeutic potential of specific microbial taxa and their associated postbiotics.

## Supplementary Material

Supplemental Material

## Data Availability

The datasets generated and/or analyzed during the current study are available from the corresponding author upon reasonable request, following anonymization.
